# Biosynthesis of Essential Polyunsaturated Fatty Acids in Wheat Triggered by Expression of Artificial Gene 

**DOI:** 10.3390/ijms161226137

**Published:** 2015-12-16

**Authors:** Daniel Mihálik, Lenka Klčová, Katarína Ondreičková, Martina Hudcovicová, Marcela Gubišová, Tatiana Klempová, Milan Čertík, János Pauk, Ján Kraic

**Affiliations:** 1 Research Institute of Plant Production, National Agricultural and Food Center, 921 68 Piešťany, Slovakia; mihalik@vurv.sk (D.M.); klcova.l@vurv.sk (L.K.); ondreickova@vurv.sk (K.O.); hudcovicova@vurv.sk (M.H.); gubisova@vurv.sk (M.G.); 2 Department of Biotechnology, Faculty of Natural Sciences, University of SS, Cyril and Methodius in Trnava, 917 01 Trnava, Slovakia; 3 Department of Botany and Genetics, Faculty of Natural Sciences, Constantine the Philosopher University in Nitra, 949 74 Nitra, Slovakia; 4 Faculty of Chemical and Food Technology, Slovak University of Technology, 812 37 Bratislava, Slovakia; tatiana.klempova@stuba.sk (T.K.); milan.certik@stuba.sk (M.C.); 5 Cereal Research Non-profit Ltd., Szeged, Alsó kikötö sor 9, H-6726 Szeged, Hungary; janos.pauk@gk-szeged.hu

**Keywords:** wheat, essential fatty acid, γ-linolenic acid, ∆^6^desaturase, artificial gene, transgene

## Abstract

The artificial gene *D6D* encoding the enzyme ∆^6^desaturase was designed and synthesized using the sequence of the same gene from the fungus *Thamnidium* *elegans*. The original start codon was replaced by the signal sequence derived from the wheat gene for high-molecular-weight glutenin subunit and the codon usage was completely changed for optimal expression in wheat. Synthesized artificial *D6D* gene was delivered into plants of the spring wheat line CY-45 and the gene itself, as well as transcribed *D6D* mRNA were confirmed in plants of T_0_ and T_1_ generations. The desired product of the wheat genetic modification by artificial *D6D* gene was the γ-linolenic acid. Its presence was confirmed in mature grains of transgenic wheat plants in the amount 0.04%–0.32% (*v*/*v*) of the total amount of fatty acids. Both newly synthesized γ-linolenic acid and stearidonic acid have been detected also in leaves, stems, roots, awns, paleas, rachillas, and immature grains of the T_1_ generation as well as in immature and mature grains of the T_2_ generation. Contents of γ-linolenic acid and stearidonic acid varied in range 0%–1.40% (*v*/*v*) and 0%–1.53% (*v*/*v*) from the total amount of fatty acids, respectively. This approach has opened the pathway of desaturation of fatty acids and production of essential polyunsaturated fatty acids in wheat.

## 1. Introduction

Wheat (*Triticum aestivum* L.) belongs to the most important crops and is an unreplaceable source of foods over the world. The most substantial components of the wheat grain intended for the human diet are saccharides (starch) and proteins, but the nutrition composed primarily of wheat and other cereal grains is not optimally balanced for the consumer from the nutritional and physiological points of view. It would be highly desirable if multiple nutritional deficiencies could be tackled using engineered crops containing high levels of different minerals and organic nutrients [1]. An example could be also wheat producing essential n-3 and n-6 polyunsaturated fatty acids (PUFAs), missing in mature grains. More than half of the amount of fatty acids in wheat grains represents linoleic acid (LA), but no γ-linolenic acid (GLA) and other essential fatty acids are present there [2]. The GLA is the main dietary essential fatty acid metabolized by the human body to a range of different and important substances [3]. Nevertheless, the human body is not able itself to synthesize linolenic acid and α-linolenic acids and these essential polyunsaturated fatty acids (PUFAs) must be obtained by food ingestion. Moreover, endogenous γ-linolenic acid (6,9,12,cis,cis,cis-octadecatrienoic acid, GLA) formation, the rate limiting Δ^6^desaturase metabolite of linoleic acid, is low or impaired in a variety of diseases and should also be supplied by diet.

Alternatively, some microorganisms can be exploited as important producers of PUFAs. Selected species of bacteria, algae, and filamentous fungi can be used in the solid state and submerge fermentation systems [4,5,6,7,8,9]. Also more than two-hundred plant species can synthesize and accumulate GLA in seeds, e.g., blackcurrant (*Ribes nigrum* L.) [10], borage (*Borago officinallis* L.) [11], and evening primrose (*Oenothera biennis* L.) [12]. Unfortunately, these species are mostly negligible from the agricultural point of view and not suitable for industrial large-scale seed and GLA production [13,14]. Nevertheless, plants including cereals generally have the objective potential to produce essential PUFAs and it can be enforced by effective techniques of relevant genes transfer. The enzyme responsible for desaturation of the linoleic acid (C18:2, n-6) to γ-linolenic acid (C18:3, n-6) is the ∆^6^desaturase (D6D) and the first isolation of the gene encoding ∆^6^desaturase was from cyanobacterium [15]. This gene was later transformed into tobacco plants [16]. Higher levels of GLA (~13%) were detected in tobacco plants transformed with the *D6D* gene originated from borage [17]. The canola plants co-transformed with the *D6D* gene and the gene encoding ∆^12^desaturase from filamentous fungus *Mortierella alpina* produced 43% of GLA within total fatty acids [18]. Expression of the *D6D* gene from *Echium gentianoides* and the GLA production were confirmed in transgenic tobacco calli [19]. Enhanced expression of GLA was reached in transgenic *Brassica juncea* plants using the seed-specific promoter from *Brassica napus* regulating the *D6D* gene from *Pythium irregulare* [20]. The seed-specific over-production of GLA in soybean transformed with the borage *D6D* gene was also reported [21]. The safflower (*Carthamus tinctorius*) transformed with the *D6D* genes originated from *Saprolegnia diclina* and *Mortierella alpina* produced abundant amounts of GLA (50%–70%, *v*/*v*) [22].

Wheat and the main cereals lack the enzyme ∆^6^desaturase responsible for catalytic conversion of linoleic acid to γ-linolenic acid [23,24] and conversion of α-linolenic acid (ALA: C18:3, n-3) to stearidonic acid (SDA; C18:4, n-3). An important prerequisite to overcome this barrier in wheat should be presence of substrates for enzyme-mediated formation of PUFAs. Changes in the biosynthesis of fatty acids in wheat and other cereals cannot be made by classic breeding methods. The existing alternative could be the synthetic biology approach combined with targeted gene transfer into plants. Using this principle the transfer of relevant genes into cereals and other crops may trigger synthesis of PUFAs and can contribute to better human health through improved nutrition [25,26].

An appropriate model for genetic transformation within cereals is rice (*Oryza sativa* L.). Improvement of PUFA content in rice seeds has been demonstrated [27] using transfer of construct contained tobacco microsome ω-3 fatty acid desaturase gene (*NtFAD3*). The content of the ALA in seeds of progenies was 2.5-fold higher than in control [28]. The first cereal seeds enriched with PUFAs up to level comparable with flax (*Linum usitatissimum* L.) seeds were also rice plants transformed with the chimeric gene consisting of cDNA of soybean microsomal ω-3 fatty acid *GmFAD3* desaturase (Δ^15^desaturase) [29]. Transgenic plants accumulated ALA up to 37.5% of the total oils in seeds. Increased amounts of ALA in rice seeds by genetic transformation using the six different ω-3 (Δ-15) fatty acid desaturase (*FAD*) genes cloned from rice and soybean and *cis*-genic modified plants contained 27.9 times more ALA while the *trans*-genic plants by the 23.8 times higher content of ALA in comparison to non-transgenic plants were reported [29]. The fatty acid desaturase genes ω-3/Δ-15 originating from rice and soybean were introduced into rice and ALA expression in embryos and bran was increased up to 27.9-fold [30]. An opposite strategy to change fatty acid composition in seeds of modified rice plants has been presented [31] and content of oleic acid in rice seeds has been increased at the expense of linoleic acid and palmitic acid, *i.e.*, suppressed formation of fatty acids with double bonds. This was achieved by suppressing the microsomal Δ^12^desaturase (*OsFAD2*) gene by RNA interference (RNAi). The expression of *FAD2*-RNAi construct was driven by the wheat’s high molecular weight glutenin promoter *Bx17*. The oleic acid content in transgenic lines has been raised to 51%–65% from the original 38%, the linoleic and palmitic acids contents were reduced. The first cereal producing the GLA in mature seeds created using the gene transfer technology was the barley (*Hordeum vulgare* L.) expressing the *D6D* transgene isolated from the filamentous fungus *Thamnidium elegans* [32].

Designing and construction of new biological parts, new biological systems, including user-designed plants can be created by synthetic biology [33]. The goal of the synthetic biology can be also to build or bypass synthetic metabolic pathways for production of novel metabolites [34]. Synthetic biology has potential for revolutionising genetic engineering of plants [35]. An understanding and manipulation of plant lipid composition can facilitate significant breakthroughs in the generation of plants with novel oils [36].

The aim of this work was to associate principles of synthetic biology and gene transfer to create the artificial gene encoding ∆^6^desaturase, transfer this gene into wheat plants, trigger novel synthetic metabolic pathway leading to the essential γ-linolenic acid and other unsaturated fatty acids in mature wheat grains, and to confirm presence of synthesized PUFAs in transgenic wheat plants.

## 2. Results and Discussion

### 2.1. Creating of Artificial Gene

**Figure 1:**
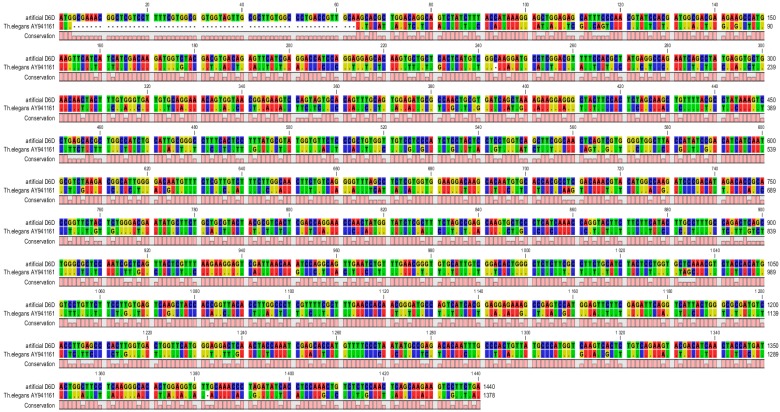
Homology in DNA sequence between artificial and original gene *D6D* from *Thamnidium* *elegans* (different colors represent different nucleotides in DNA sequences of the artificial *D6D* gene and the *D6D* gene originated from *T. elegans*, the pink line presents conformity in DNA sequences between them).

**Figure 2:**
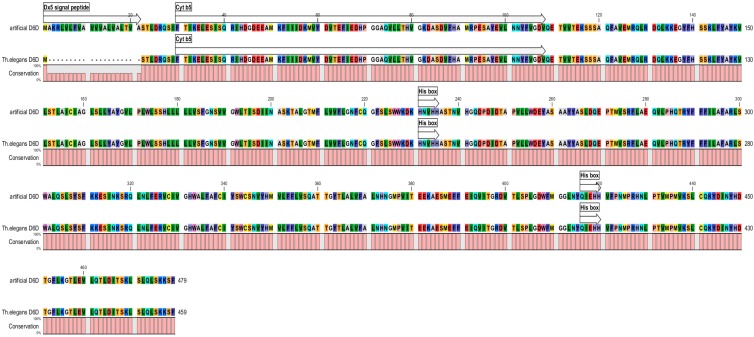
Composition of amino acids of protein encoded by the synthetic *D6D* gene and original protein from *Thamnidium* *elegans* (Dx5 signal peptide—signal sequence originated from Dx5 HMW-GS, Cyt b5—cytochrome b5 heme-binding domain; His-box—histidine rich region, boxes and arrows indicate their positions in protein sequence, different colors represent different amino acids in sequence of the artificial D6D protein and the D6D protein originated from *T. elegans*, the pink line presents conformity in amino acid sequence between them).

The artificial gene *D6D* encoding the enzyme ∆^6^desaturase was created by the synthetic biology approach. DNA sequence of the *D6D* gene from the fungus *Thamnidium* *elegans* (AY941161) was used as functional template [37]. Its original start codon was replaced by 63 base pairs long signal sequence from the wheat gene encoding high-molecular-weight glutenin subunit (HMW-GS) 1Dx5 [38] including also its start codon (Figure 1). This signal sequence should direct the synthesized protein (enzyme) to endoplasmic reticulum. Another step was the complete codon usage change for optimization of gene expression in wheat. The complete sequence of the artificial *D6D* gene was published in the GenBank^®^ database [39] HM640246. Comparison of amino acid composition of D6D enzyme encoded by the artificial *D6D* gene with original protein from the *Thamnidium* *elegans* confirmed full homology with the original enzyme, including typical cytochrome b5 heme-binding domain, and histidine rich regions (Figure 2). The signal peptide 1Dx5 was also present. The overall homology in amino acids composition between the original and artificial *D6D* genes was 71.4%.

### 2.2. Wheat Transformation by Artificial Gene

**Figure 3:**
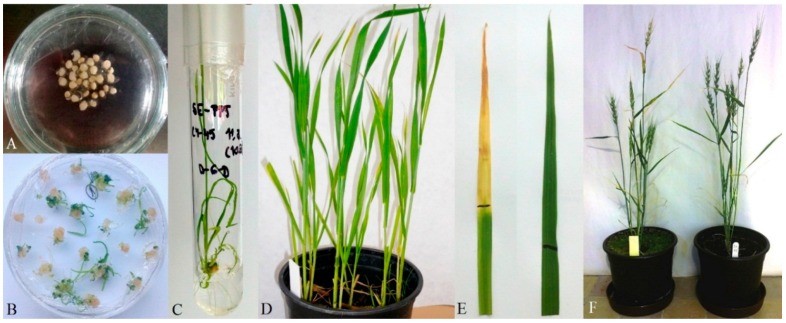
(**A**) Scutella of immature wheat embryos prepared for biolistic transformation; (**B**) Plant regeneration on selection medium; (**C**) Rooting of PPT-resistant plantlet; (**D**) Plants transferred to soil and adapted to *ex vitro* conditions; (**E**) Reaction to application of PPT on leaves (non-transformed control—left, transformed—right, black lines differentiate leaf areas with and without application of the PPT); (**F**) Mature fertile wheat plants (non-transformed control—left, transformed—right).

Five hundred immature embryos of the spring wheat (*Triticum aestivum* L.) line CY-45 were co-bombarded with the plasmids pAHC20 and pLRPT possessing also desired artificial *D6D* gene. Treated embryos formed calli and some of them regenerated in cultivation medium supplemented with the selection agent phosphinothricin (PPT). Altogether, 10 PPT-resistant plantlets were regenerated and after adaptation to *in vivo* conditions were transferred to soil and cultivated to the maturity (Figure 3).

### 2.3. Analysis of Transgenic Wheats

**Figure 4:**
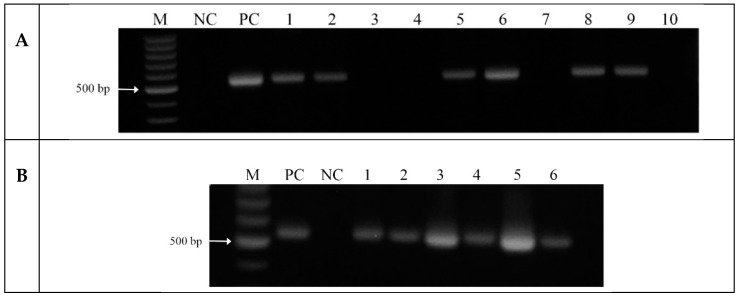
*D6D* transgene detection by PCR (**A**) and *D6D*-mRNA detection by reverse transcriptase PCR (**B**) in immature grains of T_0_ transgenic wheat plants (M—100 bp DNA marker, NC—negative control (non-transformed plant), PC—positive control, 1–10—analyzed transgenic plants).

DNA from putative wheat transformants were analyzed and the presence of artificial *D6D* transgene was confirmed in six plants of the T_0_ generation (Figure 4), each yielded 2–13 mature grains. DNA analysis confirmed that the PCR product relevant to the 517 bp fragment of *D6D* transgene was present in three of five selected T_1_ plants.

**Figure 5:**
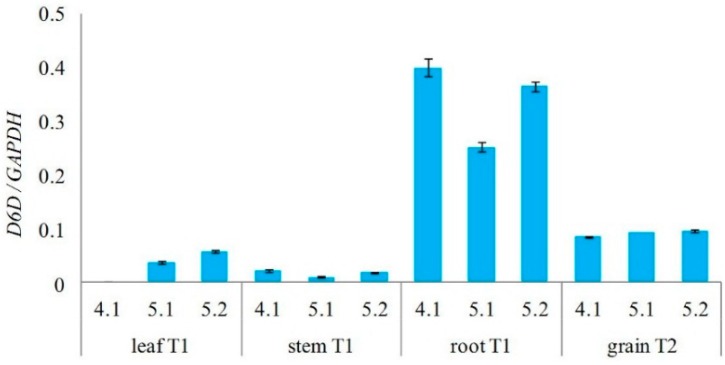
Semi-quantitative RT-PCR analysis of *D6D* transgene expression in leaves, stems, and roots of three different T_1_ plants (4.1, 5.1, 5.2) and grains of T_2_ generation. Relative amount of *D6D*-cDNA was expressed as the ratio between cDNAs of transgene and house-keeping gene (*GAPDH*). Control non-transformed plants had null values in all analyzed tissues (not shown).

Subsequently, the total RNA was isolated from *D6D* transgene positive T_0_ plants and presence of *D6D* mRNA was analyzed using the reverse transcriptase PCR (RT-PCR) (Figure 4). RNA isolated from leaves, stems, roots of the T_1_ generation, and grains of the T_2_ generation was analyzed by semi-quantitative RT-PCR to verify expression of the *D6D* transgene and determine quantity of expressed transgene product using the calibration curve in the range 2.5 × 10^−4^–2.5 × 10^3^ pg of amplified fragment. The ratio between the amount of transgene (*D6D*) cDNA and cDNA of house-keeping gene *GAPDH* (glyceraldehyde-3-phosphate dehydrogenase) was evaluated and remarkably the highest content of expressed transgene was detected in root in all analyzed plants of T_1_ generation (Figure 5). Control plants had no expression of the *D6D* transgene in all analyzed tissues.

**Figure 6:**
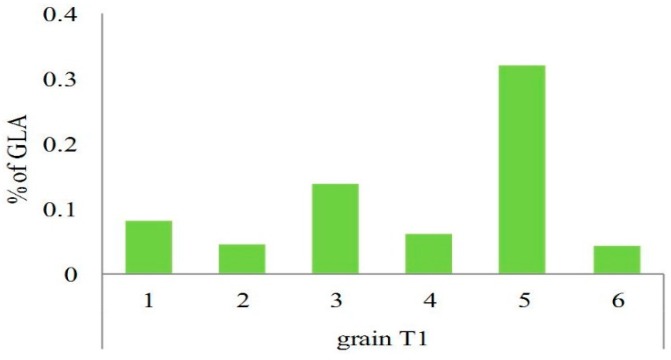
Content of GLA (percentage from the total amount of fatty acids) in grains of T_1_ generation originating from 6 transgenic plants of T_0_ generation (No. 1–6). Control non-transformed plants had null content of GLA (not shown).

**Figure 7:**
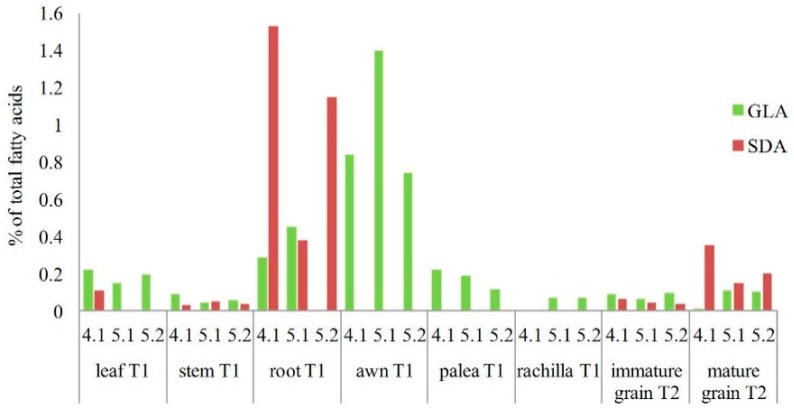
Contents of γ-linolenic acid (GLA)and stearidonic acid (SDA) in different plant tissues and grains of three transgenic plants (4.1, 5.1, 5.2) of T_1_ and T_2_ generations, respectively. Control non-transgenic plants did not synthesize both the GLA and SDA in their tissues (not included in the graph).

**Figure 8:**
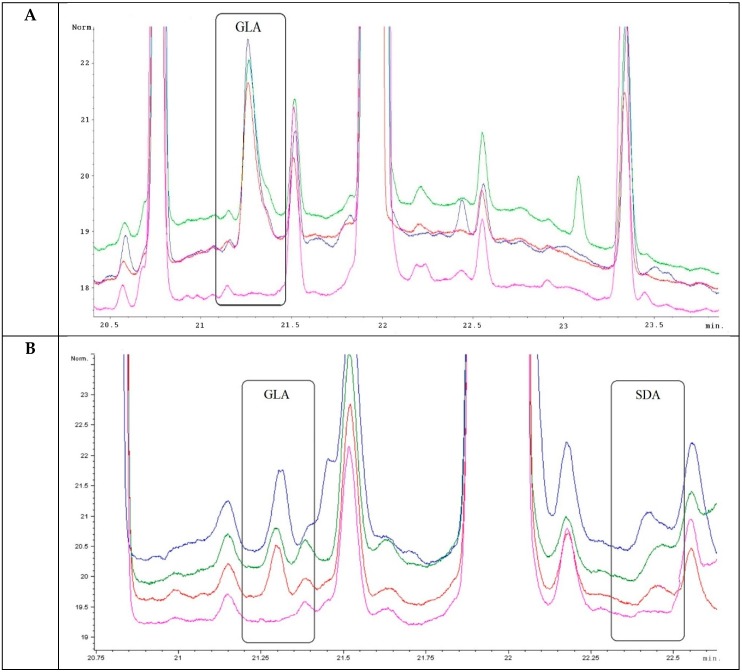
The GC-FID analysis of fatty acids composition in awns of T_1_ generation (**A**) and immature grains of T_2_ generation (**B**). The bottom most (pink colored) line represents control non-transgenic wheat plant; others (red, green, blue) lines represent transgenic wheat plants (4.1, 5.1, 5.2).

Presence of the final products of expressed artificial transgene *D6D* in the mature grains, transgenic wheat plants, and non-transformed control plants were detected by the gas chromatography equipped with a flame-ionization detector (FID) and mass spectrometer (MS). The GLA was confirmed in mature grains of T_1_ generation originating from six transgenic T_0_ plants and its content in grains varied from 0.04% to 0.32% (*v*/*v*) of the total amount of fatty acids (Figure 6). Such variation between individual transformants is the result of different transformation events that occurred in each individual transgenic plant after the genetic bombardment and after the adoption of foreign genes. Presence of the fatty acids was evaluated also in other tissues and organs (leaf, stem, root, awn, palea, rachilla) of transgenic wheat plants of the T_1_ generation and also in immature and mature grains of the T_2_ generation (Figure 7). The GLA and even the SDA were detected not only in grains, but also in other plant tissues (Figure 7). Presence of both PUFAs confirmed expression of the artificial *D6D* gene. The newly synthesized enzyme ∆^6^desaturase filled the gap in the pathway leading to GLA and SDA by utilizing of LA and ALA as substrates. Content of GLA varied in range 0%–1.40% (*v*/*v*) and SDA 0%–1.53% (*v*/*v*) of the total amount of fatty acids. The highest GLA content was detected in the awns of wheat spike and the highest SDA in roots. The immature and mature grains of T_2_ generation as well as stems, roots, and leaf samples of T_1_ generation contained both the GLA and SDA, while in all samples from different spike tissues (awn, palea, rachilla) only the GLA was present (Figure 7). The highest level of newly synthesized GLA was detected in awns of T_1_ plants (Figure 7 and Figure 8). Significant differences were observed in the formation of GLA and SDA and the ratio between them, in different organs of individual transgenic plants in T_1_ and T_2_ generation, respectively (Figure 7).

### 2.4. Analysis of PUFAs in Pathogens Invading Wheat

The reason for this part of the study was to exclude the possibility of the presence of GLA and the SDA in transgenic wheat. Both fatty acids could produce plant pathogens infecting and colonizing the transgenic wheat. Analysis of fatty acids content in common cereal pathogens using the GC-FID confirmed that the GLA and SDA which have been confirmed in analyzed transgenic wheat samples were really produced by expressed artificial *D6D* gene delivered into wheat by the biolistic transformation. On the other side, the presence of both PUFAs as a consequence of possible contamination of wheat tissues with these phytopathogens was excluded. All phytopathogen species and strains not synthesized neither GLA nor SDA and fatty acids detected within them were palmitic, stearic, oleic, vaccenic, linoleic, and α-linolenic (Table 1).

**Table 1:** Contents of fatty acids in tested cereal pathogens (PA—palmitic acid, SA—stearic acid, OA—oleic acid, VA—vaccenic acid, LA—linolenic acid, ALA—α-linolenic acid; others—fatty acids other than GLA and SDA, ND—not detected).

Pathogen	Fatty Acid (%)						
	PA	SA	OA	VA	LA	ALA	Others
*Blumeria graminis*	37.1	24.3	2.8	ND	14.0	9.2	12.6
*Drechslera tritici-repentis*	24.1	3.5	34.8	6.7	23.0	4.4	3.5
*Stagonospora nodorum*	12.3	4.7	26.0	0.9	47.8	3.6	4.8
*Septoria tritici*	27.8	6.6	31.7	0.5	22.9	3.3	7.3
*Fusarium poae*	19.9	4.2	36.4	1.2	33.0	2.0	3.3
*Fusarium culmorum*	12.7	5.3	45.9	0.8	29.9	1.3	4.2
*Fusarium graminearum*	15.0	2.3	45.9	1.5	28.9	1.6	4.9
*Alternaria* sp.	17.2	4.8	33.1	2.7	37.0	1.2	4.0

Endosperm specific promoter used in transformation experiment should direct expression of the artificial *D6D* gene and synthesis of both the GLA and SDA into the seed endosperm. The promoter 1Dx5 used in *D6D* gene transfer should maintain endosperm-specific expression especially due to its origin from the gene encoding wheat high-molecular-weight glutenin [40]. Nevertheless, both PUFAs were detected in different wheat tissues besides grains (Figure 7). Such loss of seed specificity of seed storage promoters was reported in transgenic rice and has been attributed to lower relatedness between used wheat and barley promoters and transformed rice in the *cis*-elements controlling seed specificity [41]. Generally, the organ specific promoters are in fact able to ensure expression of transgene in the embryo, endosperm, aleurone, or pericarp of seed, nevertheless expression in other tissues and organs of transgenic plant is not uncommon [42]. In addition, new factor influencing endosperm specific expression of the *D6D* transgene in our study was the artificial gene itself. Regulatory sequences, e.g., enhancers activating transcription in a tissue-specific manner are located downstream from promoter, within an intron or downstream from an exon of a gene. Just this region has been completely redesigned by synthesis of the artificial gene and their structure might have changed.

If the product of some transgene is expressing in all parts of plant body it may have negative effects on the vegetative growth of plant [42]. We can hypothesize that expression of the *D6D* transgene could induce the opposite effects. Synthesized unsaturated fatty acids could beneficially influence plant tissues, organs, and the whole body, especially at the level of biological membranes. Increased polyunsaturation of fatty acids in membrane phospholipids have been reported in many plant species as an important factor enhancing the integrity and the fluidity of the membrane relevant to increasing of chilling and freezing tolerance [43]. Generally, enhancing the tolerance of plants to temperature stresses, including high temperature, relates to membrane fluidity and is largely dictated by the composition of lipids and the degree of membrane saturation [44]. This is also the role of the *D6D* gene in *Thamnidium elegans* [37].

Our study has shown that proper principles of the synthetic biology and gene transfer technologies could be an effective way to change biosynthetic pathway of polyunsaturated fatty acids in wheat plants. Our experiment has confirmed that desaturase gene, including its artificial form, after introduction into cereal genomes can open the way for desaturation of fatty acids and synthesis of PUFAs in grains to be also considerable source of essential PUFAs for the human and animal nutrition. Production of GLA and SDA in transgenic wheat plants was relatively low, nevertheless the contribution of PUFAs to cover recommended daily energy intake is low and only several hundred of milligrams are needed in human nutrition [45].

## 3. Experimental Section

### 3.1. Biological Material

Wheat (*Triticum aestivum* L.) plants of the responsible cultivar CY-45 were cultivated in field conditions. Immature caryopses were harvested 12–16 days post anthesis, treated with 70% (*v*/*v*) ethanol for 2 min, followed by 20 min surface sterilization in commercial bleach containing 4% (*v*/*v*) of sodium hypochlorite. Grains were subsequently rinsed three times with sterile water and immature embryos were aseptically excised from the caryopses.

Common wheat pathogens of the temperate regions *Blumeria graminis*, *Drechslera tritici-repentis*, *Stagonospora nodorum*, *Septoria tritici*, *Alternaria* spp*.* were obtained from the collection of plant pathogens (Research Institute of Plant Production, Piešťany, Slovakia). Isolates of the *Fusarium poae*, *Fusarium culmorum*, and *Fusarium graminearum* were provided by Dr. Pavel Matušinský (Agrotest fyto, Kroměříž, Czech Republic). All tested phytopathogens were cultivated *in vitro* using species-specific cultivation media.

### 3.2. Artificial Gene and DNA Constructs

The DNA sequence of the artificial gene *D6D* was derived from the same gene originated from the filamentous fungi *Thamnidium elegans* published previously [37]. The original start codon of fungi was replaced by signal sequence from the gene encoding HMW-GS 1Dx5 [46] directing synthesized protein to endoplasmic reticulum. The artificial gene sequence was flanked by *Sal*I and *Xba*I restriction sites, respectively. The codon usage was completely changed and optimized for gene expression in cereal genomes using the software OPTIMIZER [47]. Designed artificial gene *D6D* was subsequently synthesized (MWG Operon, Ebersberg, Germany) and cloned into the plasmid pLRPT (provided by Dr. H. D. Jones, Rothamsted Research Station, Rothamsted, UK) along with the endosperm-specific promoter 1Dx5 originated from the HMW-GS [39] and the 35S terminator.

The plasmid pAHC20 [48] was used for co-transformation and selection of wheat transformed tissues using the herbicide phosphinotricin due to presence of the *bar* gene [49] under control of the constitutive maize ubiquitin promoter.

### 3.3. Wheat Transformation and Regeneration

Immature scutella were aseptically isolated and preconditioned on the callus induction medium supplemented with 2 mg/L of 2,4**-**dichlorophenoxyacetic acid [50] in the dark, at 25 °C, for 3–6 days. Explants were subjected to an osmotic treatment for four hours prior to particle bombardment on the induction medium supplemented with 63.7 mg/L of mannitol. Gold microparticles (3 mg) with diameter 1 µm were sonicated and coated with 5 µg of DNA in the presence of 0.1 M spermidine (20 µL) and 2.5 M CaCl_2_ (50 µL). After mixing the solution was incubated 10 min on ice and centrifuged. Supernatant was then removed and gold particles were washed with 100% ethanol and re-suspended in 100 µL of ethanol. Five microliters of suspension was used per shot.

Five hundred immature scutella (30 per plate) were bombarded with gold particles coated with plasmids pLRPT and pAHC20 (ratio amounts 3:2) simultaneously. Particle bombardment parameters were as follows: target distance 6 cm, helium pressure 1100 psi, vacuum 91.43 kPa. Transformation was performed using the biolistic particle delivery system PDS-100/He (Bio-Rad Laboratories, Inc., Hercules, CA, USA) as was described previously [51].

Day after bombardment the immature embryos were plated onto the regeneration medium supplemented with 2.5 mg/L of CuSO_4_·5H_2_O and cultivated two weeks in the dark. For induction of regeneration, the calli were exposed to 16/8 h photoperiod with light intensity 50 µmol·m^−2^·s^−1^ and temperature regime 25/20 °C, in growth regulator-free cultivation medium. Selection of transformed cells was achieved by adding of 5 mg/L of phosphinothricin (PPT) into the cultivation medium. Cultures were transferred to a fresh medium every two weeks. Recovered PPT-resistant plantlets were removed and placed into the rooting medium for an additional two to three weeks. Developed plantlets were planted in the soil, acclimatized to *ex vitro* conditions, and cultivated until maturity. Transgenic plants resistant to PPT were identified by spreading of leaves with 0.2% (*v*/*v*) of PPT solution.

### 3.4. DNA Analysis

The genomic DNA was extracted from 1 g of fresh leaves from putative transformants as well as from control plants (non-transformed) three to five weeks after transfer to soil using the DNeasy Plant Mini Kit (Qiagen, Hilden, Germany). The CLC Genomics Workbench software (CLC bio, Aarhus, Denmark) was used for primer design according to the *D6D* gene sequence (GenBank accession no. HM640246). Sequences of forward and reverse primers were 5′-GGTGGAAGGACAAGCACAAT-3′ and 5′-CGCCCAGTAATGACCTGAAT-3′. Expected size of the PCR product was 517 bp. The PCR reaction mixture (25 µL) contained: 1× PCR buffer, 1.5 mM MgCl_2_, 10 pM both of primers, 0.2 mM dNTP, 0.5 U Platinum^®^ Taq DNA polymerase (Invitrogen Corp., Carlsbad, CA, USA), and 30 ng of template DNA. The PCR was performed in a Mastercycler^®^ep (Eppendorf, Hamburg, Germany) using the following conditions: initial heat denaturation at 94 °C for 3 min, followed by 35 cycles each consisting of a denaturation step at 94 °C for 1 min, annealing at 60 °C for 45 s, extension at 72 °C for 1 min and a final extension step at 72 °C for 10 min. Products of amplification were analyzed in 1.5% (*w*/*v*) agarose gel in 1× TBE buffer (1.1% Tris-HCl; 0.1% Na_2_EDTA·2H_2_O; 0.55% boric acid) pre-stained with 0.10 µL/mL of ethidium bromide.

### 3.5. RNA Analysis

The total RNA from putative wheat transformed and control plants was extracted from 0.2 g of plant tissues and immature grains using the NucleoSpin^®^ RNA Plant isolation kit (Macherey-Nagel, Düren, Germany). Potential genomic DNA contaminants were removed by DNase treatment (Fermentas, St. Leon-Rot, Germany). Concentrations of RNA were measured spectrophotometrically (Nanodrop 1000 Spectrophotometer, Thermo Fisher Scientific, Waltham, MA, USA) and RNA quality by electrophoresis in 1.5% agarose-formaldehyde gel stained with ethidium bromide. The RevertAid First Strand cDNA Synthesis Kit (Fermentas, St. Leon-Rot, Germany) was used for the first strand cDNA synthesis. Qualitative analysis of transgene cDNA was performed by PCR with 50 ng of template. PCR conditions and composition of the amplification mixture were the same as for PCR analysis. PCR products were separated in 1% agarose gel stained with ethidium bromide.

The Real-Time PCR was carried out for artificial *D6D* gene and the *GAPDH* gene (GenBank accession No. AK359500.1) [52] used as a house-keeping gene (ABI PRISM^®^ 7000, Applied Biosystems, New York, NY, USA) with the SYBR^®^ Green dye (SYBR^®^ Green PCR Master Mix, Applied Biosystems, New York, NY, USA). Reaction mixture (25 μL) contained 12.5 μL of SYBR^®^ Green PCR master mix, 0.1 μM both of primers, 5 μL of cDNA, and water to a final volume. Primer sequences for *D6D* gene were: forward 5′-CGGGCCTTTCACTCCTTTATG-3′, reverse 5′-CCACCCCACGACTGAGTTG-3′ and for the *GAPDH* gene: forward 5′-GAAGGGCTGCTAGCTTCAACA-3′, reverse 5′-GGCCATTCCAGTCAACTTTCC-3′. Amplification of both genes was performed using the program: 50 °C for 2 min, 95 °C for 10 min, 40 cycles of 15 s at 95 °C and 1 min at 65 °C. The sizes of expected PCR products were 101 bp for *D6D* and 100 bp for *GAPDH* gene. Analyses were done in triplicate. Equal amounts of the template cDNA in two dilutions (25 and 50 ng of the total cDNA) were used in the both types of reaction mixtures. Fragments of *D6D* and *GAPDH* genes in the range from 2.5 × 10^−4^–2.5 × 10^3^ pg were used as standards for the calibration curve.

### 3.6. Fatty Acids Analysis

Fatty acids from total lipids were converted to their methyl esters [4]. The fatty acid methyl esters were analysed by gas chromatography (GC-6890 N, Agilent Technologies, Santa Clara, CA, USA) using a capillary column DB-23 (60 m × 0.25 mm, film thickness 0.25 μm, Agilent Technologies) and the FID detector (constant flow, hydrogen 40 mL/min, air 450 mL/min, 250 °C) under a temperature gradient (150 °C held for 3 min; 150–175 °C at program rate 7 °C/min; 175 °C held for 5 min; 175–195 °C at program rate 5 °C/min; 195–225 °C at program rate 4.5 °C/min; 225 °C held for 0.5 min; 225–215 °C at program rate 10 °C/min; 215 °C held for 7 min; 215–240 °C at program rate 10 °C/min; 240 °C held for 7 min) with hydrogen as carrier gas (flow 2.5 mL/min, velocity 57 cm/s, pressure 220 kPa) and a split ratio of 1/20 (Inlets: heater 230 °C; hydrogen flow 51 mL/min for 2 min, then hydrogen flow 20 mL/min; pressure 220 kPa). Peaks of the fatty acid methyl esters were identified by authentic standards of C4–C24 fatty acid methyl esters mixture (Supelco, Bellefonte, PA, USA) and evaluated by the ChemStation B 01 03 (Agilent Technologies).

## 4. Conclusions

Production of polyunsaturated fatty acids essential in human and animal nutrition would be very important in wheat grains and grains of other cereals. But, the absence of the enzyme ∆^6^desaturase in these crops, due to the absence of relevant gene, hinders desaturation of linolenic acid to γ-linolenic acid and α-linolenic acid to stearidonic acid. The idea of introduction of foreign ∆^6^desaturase gene into the wheat genome was built on the presence of substrates required for the production of GLA and SDA after their desaturation. Moreover, the principle of the synthetic biology has allowed us to synthesize the artificial gene for ∆^6^desaturase designed on the same gene of the fungus *Thamnidium elegans*. The applicability of this approach in modification of fatty acid biosynthesis towards to essential polyunsaturated fatty acids GLA and SDA was positively confirmed in the case of wheat.
